# The Feasibility of Information-Entropy-Based Behavioral Analysis for Detecting Environmental Barriers

**DOI:** 10.3390/ijerph182111727

**Published:** 2021-11-08

**Authors:** Bogyeong Lee, Sungjoo Hwang, Hyunsoo Kim

**Affiliations:** 1 Department Architectural Engineering, Dankook University, 152 Jukjeon-ro, Suji-gu, Yongin-si 16890, Korea; bglee_@dankook.ac.kr; 2 Department of Architectural and Urban Systems Engineering, Ewha Womans University, 52 Ewhayeodae-Gil, Seodaemun-Gu, Seoul 03760, Korea; hwangsj@ewha.ac.kr

**Keywords:** walkability, environmental barrier, inertial measurement unit (imu), information entropy, wearable sensing, built environment

## Abstract

The enhancement of physical activity is highly correlated with the conditions of the built environment. Walking is considered to be a fundamental daily physical activity, which requires an appropriate environment. Therefore, the barriers of the built environment should be identified and addressed. Barriers can act as external stimuli for pedestrians, so pedestrians may diversely respond to them. Based on this consideration, this study examines the feasibility of information-entropy-based behavioral analysis for the detection of environmental barriers. The physical responses of pedestrians were collected using an inertial measurement unit (IMU) sensor in a smartphone. After the acquired data were converted to behavioral probability distributions, the information entropy of each grid cell was calculated. The grid cells whereby the participants indicated that environmental barriers were present yielded relatively high information entropy values. The findings of this study will facilitate the design of more pedestrian-friendly environments and the development of diverse approaches that utilize citizens for monitoring the built environment.

## 1. Introduction

The promotion of physical activity (PA) in the general population is an essential factor that improves public health [1,2]. Numerous previous studies have shown how the built environment influences the behavior of pedestrians [3,4,5,6,7,8]. As the most widely practiced form of both transportation and PA, walking and the walking environment have been the focus of many studies [9,10,11]. In addition to environmental benefits compared to driving, walking has also been linked to diverse health benefits in terms of reducing obesity [12,13,14], improving cardiovascular health [15,16], managing diabetes [17,18], and improving one’s quality of life [19,20]. To date, several built environments have been identified that correlate to PA, including walking activity. Since walking activity is usually performed in a built environment that incorporates various elements [21], pedestrian walkability is significantly affected by the conditions of the built environment [4,6,7].

One of the methods for improving the walkability of a neighborhood is to eliminate environmental barriers as an individual’s mobility may be impeded by diverse environmental barriers. An environmental barrier, in terms of walkability, can be defined as an environmental feature that restricts the comfortable use of the built environment by an individual [22,23]. Considering that an environmental barrier is the result of the interaction between an individual’s physical capacity and the environmental demands, it can be interpreted as a relative concept [24]. For example, a physically impaired person may be uncomfortable in a built environment that is designed for a normal person. An environmental feature that causes discomfort in a certain group of pedestrians can become an environmental barrier [25,26,27].

To identify and address environmental barriers, governmental agencies have undertaken various approaches including inspection by experts and encouraging individuals to self-report [8,28]. In these approaches, inspectors observe the built environment to identify potential environmental barriers and individuals report the environmental features that can act as environmental barriers in their daily living [8,28,29,30,31,32]. Although these approaches can identify and create a list of potential environmental barriers, there may be several problems in the process of identifying these impediments. First, the inspection and self-reporting process is time-consuming and expensive, especially for extensive areas [8,31]. Second, these methods may omit certain environmental barriers as some barriers are constant objects (fixed to a specific built environment) and others are spatial-temporal objects (e.g., temporary obstacles on sidewalks, illegal parking around a crosswalk, etc.).

Recently, various sensing methods based on image processing (e.g., images, videos, and lidar) have been developed to identify potential environmental barriers [33,34,35]. Image-based approaches collect data related to the interaction of pedestrians with the built environment. Although these approaches solve the problems associated with conventional techniques in that they are not time-consuming, labor-intensive, and are not based on discontinuous monitoring, they may not be well-suited for the detection of environmental barriers. It can be considered that environmental barriers are determined based on the interactions of individuals with the built environment [8]. The environmental barrier is a relative concept as each individual has distinct characteristics, and there are various situational contexts in the built environment [23,36]. Therefore, it may be difficult to detect environmental barriers using one objective criterion [31]. Moreover, image-based methods suffer from the limitation of the line of sight [28,32]. For example, an image acquired by a camera can include more than two individuals. Given that a camera usually has one angle, more than two individuals can be overlapped in an image. Such overlaps may cause a problem in that it is often not possible to secure the line of sight required for the behavioral analysis of the obstructed individual.

As environmental barriers may cause abnormal behavior of pedestrians, the ability to capture a scene that a pedestrian interacts with to identify the environmental barrier is important. A pedestrian’s abnormal response may be the result of the interaction between the individual and an environmental barrier [32]. Fortunately, recent developments in wearable sensing technologies have shown the potential to analyze the interactions between pedestrians and the built environment [8,30,31,32,37]. Wearable sensing technologies have been used to monitor the conditions of the built environment by collecting and analyzing the physiological responses of individuals [e.g., inertial measurement unit (IMU), photoplethysmogram (PPG), electrodermal activity (EDA), electrocardiography (ECG), etc.]. These methods can capture specific features associated with a scene when an environmental barrier causes an abnormal response of a pedestrian by directly monitoring the interaction between a pedestrian’s response and the built environment.

The abnormal responses of pedestrians have been used to detect environmental barriers [8,28,31,38]. By focusing on the reason why abnormal responses occur when a pedestrian encounters an environmental barrier, the detection of the associated abnormal responses can indicate its existence. In particular, several studies have attempted to utilize gait patterns for the detection of environmental barriers [8,28,31]. In these studies, the authors assumed that human gait patterns are constant in the absence of external stimuli. As such, human gait patterns may be dispersed when there is an external stimulus such as an environmental barrier. Based on this assumption, the studies investigated whether the location of abnormal gait patterns coincided with the location of environmental barriers. The results of these investigations demonstrated the feasibility of utilizing gait patterns for the detection of environmental barriers.

Though previous studies have revealed the potential use of abnormal behavior in the detection of environmental barriers, the method of identifying this irregular response is not optimal. In previous studies, abnormality was measured based on the intensity of the responses of several individuals at a specific point [8,28,31,38,39]. Bisadi et al. [39] investigated the correlation between environmental barriers using the average of the Maximum Lyapunov Exponential (MaxLE) and heart rate values. In a study by Lee et al. [32], hot spot analysis was used based on the average of EDA values for a specific section. Kim et al. [28] attempted to identify environmental barriers based on segment-specific averages of EDA, gait pattern, and heart rate. Although these studies demonstrated the potential use of abnormal behaviors in the detection of environmental barriers, the method for determining abnormal behavior can potentially be improved. Previously reported studies have emphasized that individual behavioral characteristics are different [8,28,38,39]. As such, different individuals may respond differently to the same environmental barrier. For example, if there is an obstacle on a walking path, a pedestrian may avoid it, another individual may go over it, and so on. As previously indicated, environmental barriers may elicit different reactions depending on the individual. Thus, a measurement metric based on the diversity of individual responses to environmental barriers is required.

The purpose of this study is to develop and test a method for the identification of environmental barriers using data (response variability of individuals) collected using wearable sensors. Specifically, this study aims to: (1) develop a computational model that quantifies the various responses of individuals that can be induced by environmental barriers; and (2) investigate the feasibility of the proposed method via experimental testing.

## 2. Materials and Methods

### 2.1. Hypothesis

According to entropy theory, if one event is more likely to occur than another, then the amount of information that can be determined based on observations of that event is small [40,41]. Conversely, more information can be obtained by observing rare events [42,43]. Several approaches have been proposed to utilize the Shannon entropy or information entropy to understand the interaction between pedestrians and external conditions. Zhang et al. [44] attempted to analyze crowd safety based on the distribution uniformity. Li et al. [45] Shannon entropy based on data collected from pressure sensors to measure overcrowding conditions. Procházka and Olševičová [46] quantified emerging patterns using Shannon entropy. If we examine the amount of information (information entropy) based on the interaction between an individual’s response and the walking environment, the interaction can be understood based on the following concept.

In the absence of external stimuli, human gait tends to maintain homeostasis [47]. External stimuli disturb the homeostasis of one’s gait, which may be interpreted as different responses to stimuli [37,38]. As such, human behavior is more predictable in stable physical conditions and is less predictable when environmental barriers are present. Therefore, environmental barriers in a built environment increase the unpredictability of responses as they may cause disruptions in normal routines. For example, although most pedestrians (users) perform their normal gait on a well-maintained sidewalk, they will often modify their response in the case of a defective sidewalk. If a pedestrian recognizes an environmental barrier, he/she may slightly modify his/her path to avoid the barrier or perhaps cautiously walk over it (change his/her gait pattern). Even in instances wherein the individual does not recognize the barrier, it may affect his/her gait pattern.

### 2.2. Development of Entropy-Based Abnormality Assessment Method

In information theory, the entropy of a random variable is the average level of information or uncertainty [48,49,50]. Shannon [51] introduced the concept of information entropy to quantify the uncertainty of a random variable. The Shannon entropy (SE) can be calculated using Equation (1).

(1) $$H\left(X\right)=-\sum_{i=1}^{n}p\left(x_{i}\right)\log p(x_{i})\log p(x_{i})$$

where *H(X)* is the entropy, and *p*(*x_i_*) is the probability distribution.

To detect environmental barriers using information-entropy-based on a pedestrian’s behavioral response, it is necessary to first calculate the distribution of the response (*p*(*x_i_*) in Equation (1)) at a specific point. The pedestrian’s behavioral response can be expressed as a wide variety of features. In this study, the distribution of the responses was estimated based on the strength of the responses used in previous studies [8]. The intensity of the reaction was expressed as the sum of the three-axis acceleration acquired based on the inertial measurement unit and can be expressed as Equation (2).

(2) $$SVM_{ij}=\frac{\left[\sum_{k=1}^{n}\sqrt{x_{k}{}^{2}+y_{k}{}^{2}+z_{k}{}^{2}}\right]}{n}$$

where *n* is the total number of IMU measurements of the *j*th participant on the *i*th grid cell, *x_k_* is the kth acceleration of the anterior-posterior axis, *y_k_* is the kth acceleration of the horizontal axis, and *z_k_* is the kth acceleration of the vertical axis.

Although an signal vector magnitude (SVM) has the potential to capture a subtle bodily response [52,53,54], an SVM range varies depending on the individual because his/her physical characteristics and interaction with an environmental barrier are unique. This difference may lead to a difficultly in the integration of multiple behavioral responses. Therefore, the SVM values of each pedestrian were normalized to correct for inter-individual variance [52]. After calculating the normalized SVM values, the normalized values were classified in the range of 0.2. The total number of sections used was 40. The normalized values of gaits ranged from −3.0 to 5.0 (x-axis in a probability distribution) and each section had a range of 0.2. For example, the first section ranged from −3.0 to −2.8 (in normalized value of gait) and the last symbol ranged from 4.8 to 5.0. Moreover, to understand the range in terms of gait, it should be noted that the closer the symbol’s range is to 0 (e.g., range is from −0.2 to 0.0, and range from 0.0 to 0.2), the closer it is to normal walking. The classified values of all the subjects were sorted by location, and the sorted values were used to establish a probability distribution by location.

**Figure 1:**
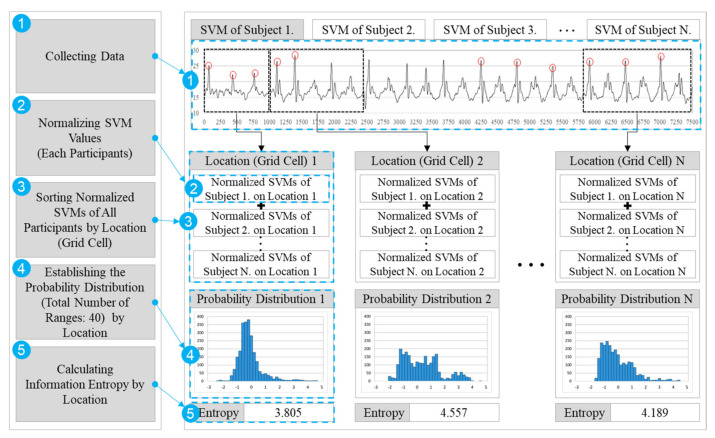
Example of the calculation process of entropy.

Figure 1 illustrates the five steps for calculating the information entropy. First, each participant’s SVM values for all the experiments were collected. Second, all the collected data of each subject were calculated as SVM values, and these SVM values were normalized. Third, the normalized SVM values were distributed to the corresponding grid cells. Fourth, the probability distribution of each grid cell was established. Finally, the entropy of each grid cell was calculated using Equation (1).

### 2.3. Experiment Design

To confirm the feasibility of identifying environmental barriers using information entropy values based on a pedestrian’s behavior, an experiment was performed in a walking environment. A total of 36 participants (20 males and 16 females) were recruited including 14 participants aged 65 years or older. None of the participants had a history of medical problems and they each voluntarily expressed their intention to participate in the experiment. Prior to the experiment, all participants were informed that the trial had been approved by the institutional review board (IRB) and that all data would be anonymized and used for research purposes only. Table 1 summarizes the demographic information of the participants.

All of the participants were asked to walk at a comfortable pace along a route established for this study (total distance of approximately 1 km). Each participant attached a smartphone to his/her waist. A fitness belt was used to affix the smartphone to the body during the experiment. During data acquisition, the smartphone collected 3-axis acceleration data and location information. To minimize the effect of changes in the external temperature or humidity, the experiment was conducted for 3 groups of 12 individuals over 3 days, from 3 September 2021 to 5 September 2021. During the experiment, the temperature was between 26 °C and 28 °C, and the humidity was between 30 and 40%.

**Figure 2:**
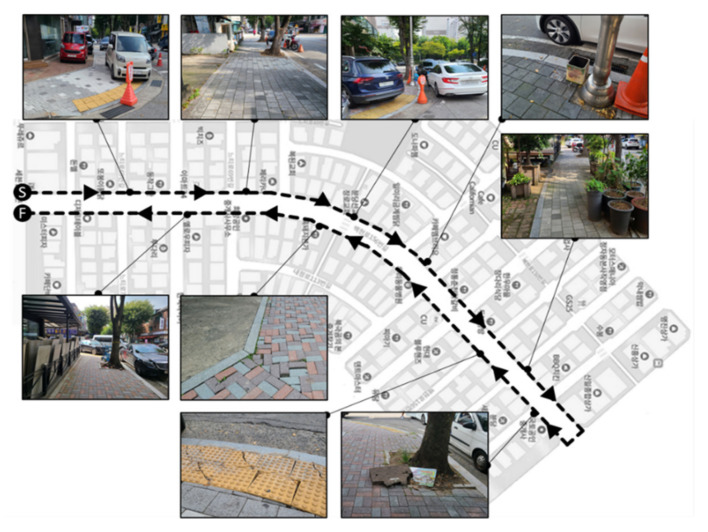
Overview of the experimental site including the path and environmental barriers.

The details of the experiment are shown in Figure 2. First, each participant listened to an introduction about the experiment for approximately 10 min at the Start Point (Point S in Figure 2) and walked along the set path to the finish point. After a break of 10 min, the experimenter and the participant walked along the path together to examine and record the points at which the participant experience discomfort or an environmental barrier. This information was used to analyze the correlation between the information entropy and the environmental barriers suggested by the participants in the data analysis process.

## 3. Results

After the participants walked along the path, it was traversed a second time with an experimenter to document the locations where discomfort was experienced, which were determined to be environmental barriers in this study. Table 2 exhibits information regarding the type and location of the environmental barriers. There are a total of 16 environmental barriers. Apart from broken blocks, which were investigated in several studies [8,29], illegal parking on sidewalks and stocked materials were also recognized as environmental barriers. The location of the environmental barriers was used for comparison with the information entropy value of each grid cell.

**Figure 3:**
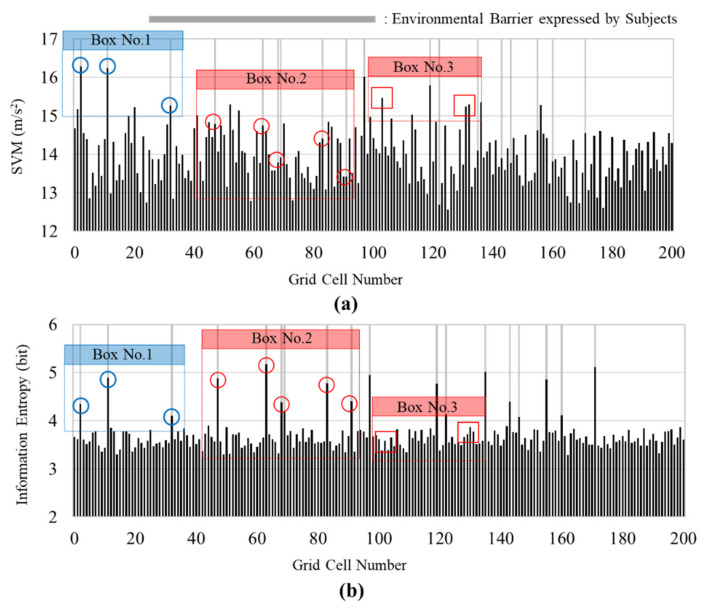
Calculation results: (**a**) Average of SVM values; and (**b**) Information entropy values.

The results of the experiments are presented in Figure 3. Each result was calculated based on the information entropy and the average of the SVM values collected from all of the participants. There are 18 grid cells marked in grey that represent the existence of environmental barriers as determined based on surveys performed in the second phase of the experiment. Figure 3a shows the SVM value of each grid cell. In several grey locations, the SVM values are higher than those of the cells which do not contain an environmental barrier. The average of the SVM values in the cells associated with environmental barriers is 14.56, and the average of the SVM values in the cells that are not associated with environmental barrier cells is 13.93. Similar to previous studies [8,28,39], this result shows an increase in the intensity of the response of the pedestrians to the environmental barriers. However, half of the 18 environmental barriers did not exhibit a significant difference in terms of the SVM values. In particular, the SVM values of cells (48, 64, 68, 70 84, 92, 136, 161, and 172) that are associated with environmental barriers do not show clear peak points. Although several cells with environmental barriers do not show a clear peak point in SVM values, there is a statistical difference between cells with an environmental barrier and cells that do not contain an environmental barrier (α < 0.05, *p* = 0.009). Therefore, they are not manifest in the data in a way that could serve as crucial information for detecting an environmental barrier in a walking environment.

Figure 3b illustrates the results for the information entropy by location. In cells that do not contain an environmental barrier, the range of the information entropy values ranged from 3.290 to 3.898, with an average of 3.606. Considering that human behavior during walking follows a regular cycle [37,38,44], the probability associated with the cells that do not contain an environmental barrier follows a normal distribution (high regularity). However, the information entropy values associated with environmental barriers are relatively high compared to those of cells that do not contain an environmental barrier. In the case of environmental barriers, information entropy values range from 4.082 to 5.176, and the average is 4.588. Moreover, all the information entropy values in the cells associated with the environmental barriers are over 4.0. In addition, a *t*-test was performed to confirm the differences between the younger and elderly groups. The significance level was set at α < 0.05, and the *p*-value was less than 0.001. Based on this result, it can be confirmed that the difference between the two groups is statistically significant.

Comparing the SVM value and the information entropy value in more detail, the following three interesting points can be observed. First, both the peak value of the SVM and the peak value of information entropy coincide with the environmental barrier at Box No. 1 in Figure 3. However, in Box No. 2, the SVM values in the cell corresponding to the environmental barrier are not represented as peak values. However, in the case of information entropy, the peak values and the existence of environmental barriers coincide. The SVM values do not exhibit peak values even when the behavior of pedestrians changes due to the environmental barriers because their response is diverse. Some participants displayed a stronger reaction (high magnitude of the response) to an environmental barrier; others displayed behaviors including walking cautiously in response to external stimuli. In this case, the average SVM value at a point may not coincide with the value associated with the existence of an environmental barrier. Although the SVM values in Box No.2 do not coincide with the existence of environmental barriers, the peak points of information entropy and the existence of environmental barriers coincide. As the variability of the response increases, the irregularity increases. As such, as the amount of information increases, the value of the information entropy increases. Finally, the points identified by red rectangles for Box No. 3 in Figure 3a show higher SVM values than the average value despite the absence of environmental barriers. Conversely, the points identified by the red rectangles in Box No. 3 in Figure 3b are within the range of information entropy values for cells that do not contain an environmental barrier.

To confirm the feasibility of using information entropy to detect environmental barriers, the relationship between each calculated value (SVM and information entropy based on the presence or absence of environmental barriers was quantitatively compared. The SVM and information entropy values of the cell are continuous variables, whereas the existence of environmental barriers can be represented as a binary variable (existence as 1 and nonexistence as 0). To investigate the relationship between the existence of an environmental barrier and the response of the participant in a statistical manner, this study used the point biserial correlation coefficient. The point biserial correlation coefficient is generally used when one variable is dichotomous and the other is continuous. For this coefficient, the values that relate to the existence of the environmental and the pedestrian’s response were calculated using Equation (3) as follows:

(3) $$r_{pb}=\frac{\left(M_{1}-M_{0}\right)\sqrt{\left(n_{1}n_{0}/n^{2}\right)}}{\sqrt{\frac{1}{n}\overset{n}{\underset{i=1}{\sum }}{\left(X_{i}-\bar{X}\right)}^{2}}}$$

where *r_pb_* is the point biserial correlation coefficient; *M*_1_ is the mean value of the continuous variable *X* (SVM values or information entropy values) for all data points in group 1 (existence of environmental barrier); *M*_0_ is the mean value of the continuous variable *X* (SVM values or information entropy values) for all data points in group 2 (nonexistence of environmental barrier); *n*_1_ is the number of data points in group 1; *n*_0_ is the number of data points in group 2, and *n* is the total sample size.

Based on the point biserial correlation, the correlation between each metric and the existence of an environmental barrier was compared. The coefficients of each metric were 0.254 (α < 0.05, *p* = 0.002) for the SVM values and 0.842 (α < 0.05, *p* < 0.001) for the information entropy values. According to previous studies [49,50], a correlation coefficient over 0.7 indicates a high degree of correlation. In the results, it was determined that the information entropy (of a pedestrian’s collective response) and the existence of environmental barriers are highly correlated. The correlation of SVM is much lower than the correlation of information entropy. As previously mentioned, the SVM values of cells (number 48, 64, 68, 70 84, 92, 136, 161, and 172 in Figure 3a) do not match well with the existence of environmental barriers. Upon comparing the information entropy values are well matched with the existence of environmental barriers, the more unmatched points of the SVM decrease the coefficient of correlation. As previously indicated, environmental barriers can be partially identified only by the intensity of the pedestrian’s behavior (SVM value). However, it can be experimentally confirmed that the value of the information entropy, which considers that pedestrians have a diversity of responses owing to the characteristics of the environmental barrier, can be more effective for detecting environmental barriers.

## 4. Discussion

### 4.1. Effectiveness of Data Collection from Diverse Groups

**Figure 4:**
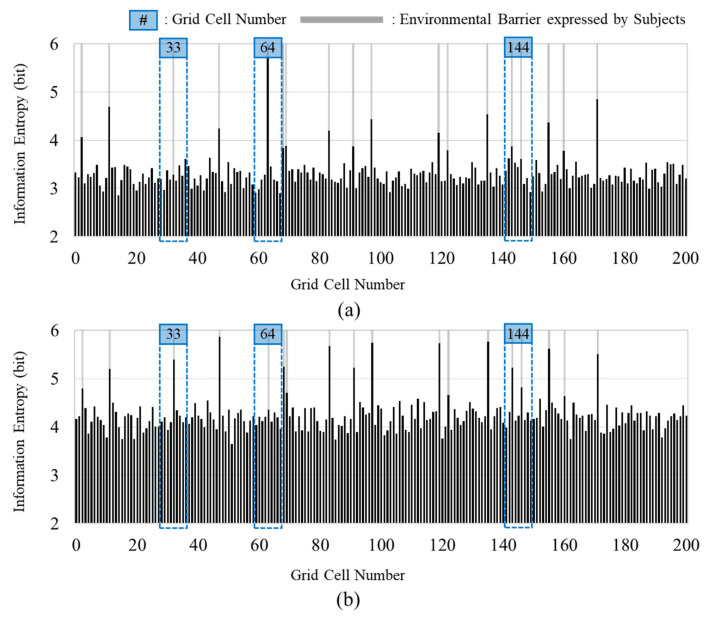
Information entropy by group: (**a**) Younger group; and (**b**) Elderly group.

In this study, 22 individuals in their 20’s to 30’s (the younger group) and a second group with members over 65 years (the elderly group) were recruited. Figure 4 shows the information entropy values of each group according to location. Overall, the younger group and the elderly group show similar plots. However, the average values of the 2 groups are 3.331 (the younger group) and 4.268 (the elderly group). Apart from the similarity of the patterns, the average values of the information entropy of these two groups show a clear difference. In particular, several cells show different patterns of information entropy between the two groups for the same environmental barrier, as indicated by the three blue boxes in Figure 4. In cell no.33, motorcycles were illegally parked on the sidewalk. The elderly group show a relatively high information entropy value although the space on either side of the motorcycle (approximately 80 cm) was sufficiently wide for a pedestrian to pass through. Second, there was an illegal smoking area in cell no. 64. When the experiment was conducted, smokers were temporarily present. A smoker was in the cell when six of the younger group passed that cell during the experiment. Although it was a temporary situation, the six younger participants appeared to have exhibited unusual behavior in the process of avoiding the smoking area. For this reason, it appears that a high information entropy value was observed for this cell. Finally, in cell 144, the pavement blocks of the pedestrian path were not fixed. Although the area of broken and unfixed blocks was not large, it was determined that they served as an environmental barrier that could induce significant behavioral changes in the elderly group. In addition, a t-test was performed to confirm the differences between the younger and elderly groups. The significance level was set at α < 0.05, and the *p*-value was less than 0.001. Based on this result, it can be confirmed that the difference between the two groups is statistically significant. A comparison of the information entropy values of the two groups indicates that the data collected from various groups can assist in the detection of additional potential environmental barriers.

### 4.2. The Possibility of Wearable-Based Sensing Approaches for Detecting Environmental Barriers

Built environmental monitoring and assessment is usually conducted by experts or trained inspectors from governmental agencies. Despite the importance of maintaining sufficient functions and conditions of the built environment for citizens, the time interval between inspections may be long depending on the availability of staff and funding. For this reason, continuous monitoring of the built environment is rarely performed. The recent development of wearable sensing devices and the innovation of people-centric sensing is expected to not only solve existing problems, but also provide new opportunities. If the users of a built environment use wearable equipment and supply data acquired during daily activity, this can serve as the basis for monitoring. The information-entropy-based approach proposed in this study can provide information on the interaction between citizens and environmental barriers. In particular, this study suggests a method wherein data can be continuously collected and utilized data to identify environmental barriers using smartphones. The proposed approach can identify a location using an entropy value, which facilitates identification based on continuous monitoring. Despite these advantages, several challenges must be addressed to improve the feasibility of the approach, including the recruitment and sampling of participants [55], and the protection of their privacy [56].

### 4.3. Contibutions of the Proposed Method

The information entropy-based environmental barrier detection method proposed in this study hypothesizes that pedestrians’ responses become more irregular when environmental barriers are present. This hypothesis was confirmed through the results of the experiments. Information entropy is a metric used to confirm the existence of environmental barriers. When there is an environmental barrier, pedestrians exhibit various reactions in response to the barrier. Information entropy based on the diversity of behaviors is highly correlated with the existence of environmental barriers compared to existing intensity-based approaches. The reason for this result can be inferred as follows. First, responses due to individual physical and cognitive differences are diversified, and information entropy is a metric that can represent this diversity well. In addition, information entropy is the average of all the available information. That is, various responses to the external environment mean that the amount of information increases, which in turn implies that even if only some of the pedestrians show irregular responses, they are included in the information entropy calculation process. Therefore, information entropy can be used as a metric to find the discomfort groups (e.g., the elderly, children, disables, etc.) that may be vulnerable to environmental barriers. Therefore, it can be observed that the information entropy of pedestrians has high utility for environmental barrier detection. In addition, the method proposed in this study can enable citizens to play the role of data providers in future smart cities. In other words, a citizen with a smartphone generates data during the walking process, and the server analyzes the data to automatically identify environmental barriers. This analysis can be performed in near-real-time if the server has sufficient data transmission/reception and processing capabilities.

### 4.4. Limitations and Future Research

This study focused on investigating the feasibility of utilizing information-entropy-based behavioral analysis for detecting environmental barriers. Although the results indicate that the suggested approach is feasible, there may be several limitations related to its real-world application such as diverse pedestrians and their behavior, and complex environments. In this study, the recruited participants were healthy individuals who had no discomfort in walking. However, some of the participants may have been more sensitive to the conditions of the walking environment or the presence or absence of environmental barriers than others. In particular, it was necessary to consider children or individuals who required assistive devices (e.g., wheelchairs, canes, walkers, etc.) because their ambulatory characteristics are different from those of normal healthy adults. Second, the walking environment that was investigated in the study did not effectively represent a typical real-world environment. In the real world, not only the fixed walking environment, but also the non-fixed walking environment must be considered. For example, there are factors such as the movement of vehicles or other pedestrians in the walking environment. In this study, the temporary environmental barriers such as the smokers in cell No. 64 may be considered as a time-dependent factor. Moreover, privacy issues must also be resolved for the general application of the proposed approach. In this study, the authors collect data from people’s daily lives and identify environmental barriers. In this process, the travel route, length of stay, and physical activity can be examined via the individual’s daily life data, and as a result, the potential infringement of privacy must be considered. In the investigation, the authors manually handled and anonymized all the data collected from the participants. Once the suggested approach is practically applied to a broad extent such as at the city level, such a manual anonymization process may not be possible. Thus, data processing for anonymization and encryption will be investigated in the future.

## 5. Conclusions

Environmental barriers in the walking environment may cause discomfort to pedestrians or serve as a factor that hinders physical activity. Therefore, environmental barriers in a built environment should be identified and addressed. However, current practices such as surveys and inspections by experts are usually time-consuming and labor-intensive. Moreover, they are typically not continuous. To address these issues, the feasibility of information entropy using data obtained via wearable sensors was investigated to detect environmental barriers. In this respect, it was hypothesized that there is a relationship between information entropy and environmental barriers. To test this hypothesis, 36 participants were recruited, and the participants participated in an experiment that involved the attachment of a smartphone to their body. After data collection, the probability distribution of the gait data for each grid cell was obtained and the information entropy was calculated. The environmental barriers and information entropy obtained at the 1 km site showed a high correlation. The findings indicate that information entropy can be an effective metric for the identification of environmental barriers.

The main contribution of this paper is the confirmation of the correlation between the existence of an environmental barrier and the associated information entropy value owing to the response of a pedestrian. In particular, an environmental barrier acts as external stimuli that can hinder a pedestrian’s normal gait. Moreover, the wearable-sensor-based approach utilized in this study facilitates continuous and facile monitoring of the walking environment. This approach can be extended to serve as the basis of people-centric sensing and participatory sensing for the improved monitoring of a built environment.

## Figures and Tables

**Table 1:** Summary of participants’ information.

Statistical Parameter	Age	Height (cm)	Weight (kg)
Mean	42.28	170.86	70.63
Median	31	171	68.94
Standard Deviation	20.87	8.07	11.51
Maximum	70	183	90.48
Minimum	20	158	48.76

**Table 2:** Environmental barriers at the experimental site.

Cell #	Description	Figure	Cell #	Description	Figure
3	Broken blocks	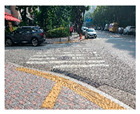	98	Obstacle	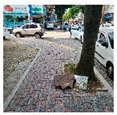
12	Parked vehicles with narrow path	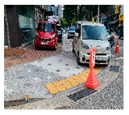	120	Broken blocks	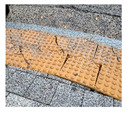
33	Parked vehicles with narrow path	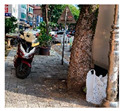	123	Parked vehicles with narrow path	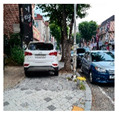
48	Parked vehicles (narrow path)	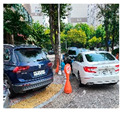	136	Parked electric scooter	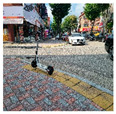
64	Illegal smoking area	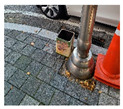	144	Broken and unfixed blocks	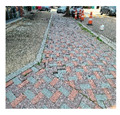
69	Unfixed blocks	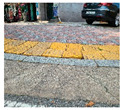	147	Illegally stocked materials	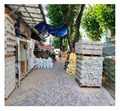
70	Illegal smoking area	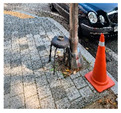	156	Parked vehicles with narrow path	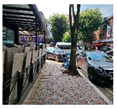
84	Stocked materials	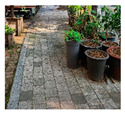	161	Illegally parked bicycle	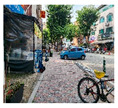
92	Trash	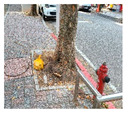	172	Unfixed manhole	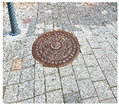

## Data Availability

Some or all data, or code generated during the study are proprietary or confidential in nature and may only be provided with restrictions (e.g., anonymized data).
